# Evaluation of intravenous regional anaesthesia and four-point nerve block efficacy in the distal hind limb of dairy cows

**DOI:** 10.1186/s12917-017-1250-x

**Published:** 2017-11-07

**Authors:** S. Yavari, N. Khraim, G. Szura, A. Starke, E. Engelke, C. Pfarrer, K. Hopster, M. Schmicke, W. Kehler, M. Heppelmann, S. B. R. Kästner, J. Rehage

**Affiliations:** 1 Clinic for Cattle, University of Veterinary Medicine Hannover, Foundation, Hannover, Germany; 2 Department for Veterinary Surgery, College of Veterinary Medicine, An-Najah National University, Nablus, Israel; 3 Clinic for Ruminants, University of Leipzig, Leipzig, Germany; 4 Institute for Anatomy, University of Veterinary Medicine Hannover, Foundation, Hannover, Germany; 5 Clinic for Horses, University of Veterinary Medicine Hannover, Foundation, Hannover, Germany; 6 Clinic for Small Animals, University of Veterinary Medicine Hannover, Foundation, Hannover, Germany

**Keywords:** IVRA, NBA, Cattle, Hind limb, Nociception

## Abstract

**Background:**

Intravenous regional anaesthesia (IVRA) and hindfoot four-point nerve block anaesthesia (NBA) are recommended for local anaesthesia (LA) in the distal limb of dairy cows. Two studies were conducted to compare the efficacy, time until onset and stress responses to IVRA and NBA in dairy cows. In the first cross-over designed study, eight healthy unsedated German Holstein cows, restrained in lateral recumbency (LR) on a surgical tipping table, were treated with IVRA and NBA using procaine 2% as a local anaesthetic. Distal limb desensitization was tested by electrical (e-), mechanical (m-) and thermal (t-) nociceptive stimulation 10 min before and 15 and 30 min after LA. Hormonal-metabolic (blood concentrations of cortisol, lactate, non-esterified fatty acids, and glucose) and cardio-respiratory (heart and respiratory rate, mean arterial blood pressure) stress responses to treatment were assessed at predetermined intervals. In the second study, six healthy, unsedated German Holstein cows in LR were treated (crossover design) with IVRA and NBA. Short-interval e-stimulation was measured by the time until complete distal limb desensitization.

**Results:**

In the first study, four of eight cows responded to e-stimulation 15 min after IVRA, while none of the cows treated with NBA responded until the safety cut-off level was reached. E-stimulation revealed complete desensitization of the distal limb 30 min after LA in all cows. Half of the cows did not respond to m- and t-stimulation before LA, so no further evaluation was performed. Stress reactions to IVRA and NBA treatment were similar, but differences may have been masked by stress response to LR restraint. In the second study, complete desensitization was achieved 12.5 min after NBA, while one of the six cows still responded to e-stimulation 20 min after IVRA.

**Conclusion:**

Hindfoot nerve block anaesthesia and intravenous regional anaesthesia induced complete desensitization of the distal hind limb in dairy cows. However, the anaesthesia onset after NBA was significantly faster than that of IVRA, which may be clinically relevant in the field, particularly when distal limb anaesthesia is required for major claw surgeries under time constraints.

## Background

Claw diseases are frequent health disorders in dairy cattle that are mainly caused by claw horn lesions, such as sole ulcers and white line disease, or by inflammatory alterations of the adjacent soft tissue [1–5]. Claw horn lesions are commonly treated in their early stages by therapeutic claw trimming [6–8]. Major surgical interventions, such as claw amputation or resection of the distal interphalangeal joint, are often necessary in advanced cases when inflammatory purulent alterations penetrate the pododerma and reach the inner structures of the horn shoe, resulting in purulent arthritis of the distal interphalangeal joint, osteomyelitis or necrosis of tendons and ligaments [9, 10]. Claw surgeries are painful for affected cows and require adequate pain management including local anaesthesia (LA) and analgesic application to control postoperative pain [11–16].

For LA at the distal limb, intravenous regional anaesthesia (Bier block [17, 18]; IVRA) or hindfoot nerve block anaesthesia (NBA) are recommended [9, 13, 19–21]. For IVRA of the hindfoot, a tourniquet is placed around the metatarsus to interrupt blood flow before local anaesthetics are injected into a vein to desensitize the limb distally to the tourniquet [18, 19]. Hindfoot nerve blocks require perineural injection of local anaesthetics to the appropriate branches or continuations of the fibular and tibial nerves [20, 21]. Although lameness is a significant health problem in cattle, treatment recommendations for claw lesions are mostly based on “experience” and “anecdotal” reports in journals or in textbooks. Few controlled studies have been reported in peer-reviewed journals [22]. To the best of the authors’ knowledge, this also includes LA techniques to the distal limb in cattle.

An advantage of the IVRA tourniquet is reduced intraoperative bleeding, which improves the surgical field visibility. However, as in humans, the tourniquet can cause traumatic nerve injury and ischaemia, which can lead to tissue damage and increased risk of infectious surgical complications after prolonged application times [23, 24]. In humans and horses, both placement and removal of a tourniquet induce moderate pain [23–25].

Due to the lack of literature on this subject, this study compared IVRA and NBA in dairy cows in terms of their efficacy, time until onset and hormonal-metabolic and cardio-respiratory stress responses.

## Methods

After ethical review, the Office of Consumer Protection and Food Safety of the Federal State of Lower-Saxony, Germany (Ethics Committee for Animal Experiments; file number: 33.19–42,502–04-1,511,970) approved the study. All experimental cows used in the study were owned by the Clinic for Cattle of the University of Veterinary Medicine Hannover.

### Study 1

#### Experimental animals and study design

In a crossover designed study, eight non-lactating, non-pregnant, healthy and unsedated German Holstein Friesian dairy cows (aged 3–6 years) were used to compare the efficacy of anaesthesia, as well as the hormonal-metabolic and cardio-respiratory stress responses, after IVRA and NBA (8 cows × 2 treatments). The washout period was two weeks. Four cows received IVRA first and two weeks later NBA and in four cows the order of treatments was vice versa. Experimental animals were housed together in a straw yard and fed a diet of grassland hay and 1 kg of concentrate for maintenance with ad libitum access to fresh water. To apply IVRA and NBA, test for anaesthesia efficacy, and evaluate the stress responses to anaesthesia, cows were positioned in left lateral recumbency (LR) on a hydraulic surgical tipping table padded with a rubber-foam mattress (Werner GmbH, Höhenkirchen, Germany).

#### Local anaesthesia at the distal hind limb

##### Intravenous regional anaesthesia (IVRA)

In all cows, IVRA was administered in the right distal hind limb using the common dorsal digital vein for aseptic intravenous administration of local anaesthetics [9, 18, 20, 26, 27]. The injection site was washed, shaved, and degreased with medical alcohol and disinfected with iodine. A trained surgeon (N.K.) placed a rubber tourniquet (Esmarchbinde, 6 cm width, 1.1 mm thickness, Wirtschaftsgenossenschaft deutscher Tierärzte, Garbsen, Germany) circumferentially in the middle of the metatarsus and punctured the vein distally from the tourniquet with a hypodermic needle (1.0 × 40 mm, Henry Schein, Gillingham, UK). Under gentle flexion of the fetlock joint, blood was drained through the needle until the pressure dropped as indicated by slow dripping rather than blood running out of the hub. Twenty millilitres of procaine hydrochloride 2% (Procasel 2%, Selectavet, Weyarn-Holzolling, Germany) were injected slowly. The injection site was compressed with an iodine soaked cotton swab for 30 s after removing the needle to avoid unintended drainage of the local anaesthetic from the punctured vein or formation of a haematoma.

##### Hindfoot nerve block anaesthesia (NBA)

For the slightly modified technique of hindfoot four-point nerve block anaesthesia according to Raker [28] in the right hind limb injection sites were surgically prepared as described above. Procaine (Procasel 2%, Selectavet) was injected perineurally (hypodermic needle, 0.8*40 mm, Henry Schein) next to the medial and the lateral plantar nerves (each 10–15 ml), both continuations of the tibial nerve (at approximately the middle of the metatarsus, between the suspensory ligament (interosseous m.) and the flexor tendons on the medial and lateral aspect of the limb). Anaesthesia of the superficial and deep fibular (peroneal) nerves was little more proximal than described by Raker [28]. Perineural injections of about 15 ml procaine anaesthetized the superficial fibular nerve (subcutaneously, latero-dorsally, 2 cm proximally to the tarsocrural joint) and of about 10 ml the deep fibular nerve (dorso-medially in the proximal third of the metatarsus, between the tendons of the long digital extensor and the metatarsal bone, in the longitudinal sulcus of the metatarsus).

##### Testing of distal limb desensitization

Desensitization was tested at room temperature (21 °C) in LR by means of electrical, thermal and mechanical stimulation. Stimulation was stopped and the actual electrical, thermal, and mechanical nociceptive threshold was recorded as soon as the cows expressed aversion to the stimulation as indicated by attempts to withdraw the leg, claw movements, muscle twitching or when the cut-off value was reached.

##### Electrical nociceptive stimulation

For constant current (CC) electrical nociceptive stimulation [29] a commercial nerve stimulator (Grass S48, CCU1; Grass Medical Instruments, MA, USA) was used as previously described [30]. At the lateral coronary band of the lateral claw of the right hind limb, two surface electrodes (Neuroline® 70,005-J/12, Ambu GmbH, Bad Nauheim, Germany) were attached to the shaved, cleaned, dried and degreased skin with an inter-electrode distance of two cm. Skin resistance measured before stimulation was always below 3 kΩ (digital multimeter, VC260, Voltcraft, Conrad Electronic SE, Germany). Noxious stimuli consisted of a 25 ms train of five 1 ms CC square-wave pulses. The trains of five were delivered at a frequency of 5 Hz. The voltage was continuously increased (5 V/s) until a maximum voltage of 150 V. For statistical evaluation, mean values of duplicate tests (2 min intervals) were used.

##### Thermal nociceptive stimulation

Thermal nociceptive stimulation was performed by means of the Thermal Threshold Testing System (TT1) (Topcat Metrology Ltd., Ely, Cambridgeshire, UK) with a safety cut-off set to 55 °C and a heating rate of 0.6 °C/s as previously described for horses [31, 32]. A 5 g thermal probe was attached to the shaved, cleaned, dried, and degreased skin at the lateral coronary band of the lateral right claw and was fixed with an elastic bandage (Vetrap, width 10 cm; Wirtschaftsgemeinschaft deutscher Tierärzte) to guarantee constant skin contact with the thermal probe. The thermal probe was equilibrated with the skin temperature for 5 min.

##### Mechanical nociceptive stimulation

For mechanical nociceptive stimulation, a handheld Medio-Line 40,025 (Schumann GmbH, Sillerup, Germany) with a blunt tip of 1.7-mm diameter and a safety cut-off set at 20 N was used. The force was increased with a rate of 1 N/s until the animal had an aversive reaction [33]. Stimulation was applied laterally and medially to the flexor tendons, approximately 5 cm above the dew claws, at the soft skin immediately proximal to the lateral and medial heel bulb of the right lateral claw, and medio- and latero-dorsally of the first phalanx. Nociceptive testing included two consecutive mechanical stimulations with 2 min inter-stimulation intervals.

##### Macroscopic examination of the skin at nociceptive stimulation sites

Before the cows were returned to standing from LR, the adhesive electrodes and thermal probe were removed. The skin was examined for alterations, visually and by palpation (reddening, swelling, lesions), where the electrodes and thermal probe were located and at the site of mechanical stimulation.

#### Evaluation of cardio-respiratory, hormonal, and metabolic stress responses

##### Instrumentation

One hour before the start of the experiment, cows received indwelling catheters under aseptic conditions into their right jugular vein (Stericlin®, 2.4 × 200 mm, Walter Veterinär-Instrumente e.k., Baruth/Mark, Germany) and their right auricular artery (Vygon, 20G–L, Leader-Flex, Ecouen, France) as previously described [34]. For placement, cows were fixed with a head halter and two abdominal belts to the surgical table in standing position.

##### Cardio-respiratory stress responses

A patient monitor (IntelliVue MP50; Philips Medizin Systeme, Germany) continuously recorded mean arterial blood pressure (MAP; IntelliVue MP50; Philips Medizin Systeme, Germany) after connection of the auricular artery catheter to a pre-calibrated fluid-filled pressure transducer (Smith pvb, REF ST-37, Critical Care GmbH, Germany) [35]. The level of the shoulder joint was considered as the zero pressure point in standing cows, and the centreline of the sternum was the reference point during LR. The auricular artery catheter was continuously flushed (3 ml/h) with heparinized saline solution [34]. The patient monitor also recorded the heart rate (HR). The respiratory rate (RR) was determined by counting thoracic expansions for a period of 1 min.

##### Hormonal and metabolic stress responses

Venous blood samples were collected in predetermined intervals from the jugular vein catheter and centrifuged at 1500 x *g*. Serum from tubes without anticoagulant (Sarstedt, Nümbrecht, Germany) and plasma from Li-Fluorid tubes (Sarstedt) were stored at −80 °C until analysis. Plasma concentrations of glucose and lactate, and serum concentrations of non-esterified fatty acids (NEFA), were measured using commercial test kits on an automated analysing system (ABX Pentra 400, Moriba ABX Diagnostics, Montpellier, France), and serum cortisol concentrations were measured by a commercial chemiluminescent enzyme immunoassay (Siemens Diagnostics, Germany).

#### Experimental protocol of study 1

To apply LA, test distal limb desensitization by nociceptive stimulation, and record hormonal-metabolic and cardio-respiratory stress responses to LA, cows were restrained for approximately one hour in lateral recumbency on a surgical tipping table without sedation [34]. During LR front legs remained loose and hind limbs were fixed with a shackle (5 cm width) at the metatarsus to a foam cushion (7 cm height, 10 cm width and length) padded leg halter. The IVRA tourniquet was removed approximately 8 min after the cows were returned to standing position.

Cows were stimulated (electrical, thermal, and mechanical nociceptive stimulation) 10 min before and 15 min and 30 min after LA.

Hormonal-metabolic and cardio-respiratory stress responses to local anaesthesia (5 min before, and 2, 14, 29, and 40 min after LA) and to restraint in LR (20 min and 5 min before and 7 min after restraining) were studied in predetermined intervals. After cows were returned to standing stress responses to removal of the tourniquet were recorded in IVRA cows (5 min before and 10 and 20 min after removal of the tourniquet) and in NBA cows (no tourniquet application) at corresponding time points.

### Study 2

#### Experimental animals and study design

Six healthy, non-lactating, non-pregnant and unsedated German Holstein cows (aged 4–6 years) were used in a cross-over design (6 cows × 2 treatments) to evaluate the time until onset of anaesthesia after IVRA and NBA by electrical nociceptive stimulation. Cows were fixed in left LR on a surgical tipping table for LA and nociceptive stimulation at the right hind limb.

Electrical nociceptive stimulation electrodes were placed on the dorso-lateral coronary band and on the soft skin immediately proximal to the lateral heel bulb of the right lateral hind claw. Electrical stimulations were performed as described above. Cows were stimulated five minutes before, and 5 min, 7.5 min, 10 min, 12.5 min, 15 min, and 20 min after LA.

#### Statistical evaluation

Statistical evaluation was performed using the Statistical Analysis System package (SAS version 9.3 for Windows, SAS institute Inc., Cary, NC, USA). Results of the nociceptive electrical stimulation and stress response to anaesthesia, change in body position, and tourniquet removal were evaluated by 2f–ANOVA in a general linear model (Proc GLM) with repeated statements (factors: group [1: IVRA, 2; NBA], time, group*time). The paired *t*-test was used to evaluate differences between mean values at specific time points within groups compared to baseline values. Group differences in the frequencies of non-responses to maximal electrical nociceptive stimulation were determined by Fisher’s EXACT Test (Proc freq) for significance. The significance level was set at *p* < 0.05.

## Results

### Study 1

#### Nociceptive stimulation

##### Electrical nociceptive stimulation

**Fig. 1:**
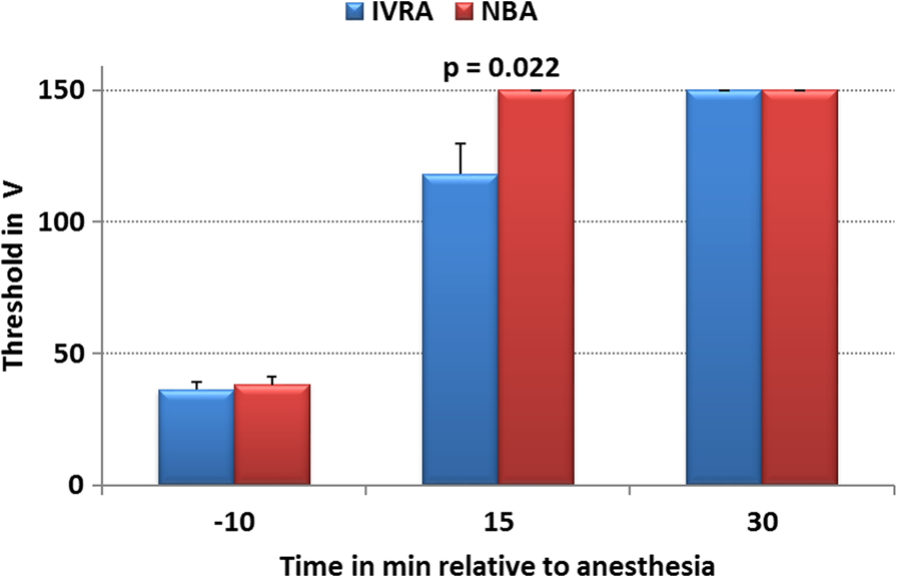
Mean thresholds (mean ± SEM) of electrical nociceptive stimulation at the lateral coronary band of the lateral claw of the right hind limb until an aversion response was noticed in dairy cows ten minutes before and 15 and 30 min after intravenous regional anaesthesia (IVRA; *N* = 8) and hindfoot nerve block anaesthesia (NBA; N = 8; study 1). Cut-off of electrical stimulation was 150 V (ANOVA effects: group: *p* = 0.033, time: *p* < 0.001, group x time: *p* = 0.019; significant group differences in mean values at specific time points are indicated by *p*-values within graph)

Before LA in both groups, the mean threshold was 36.4 ± 8.8 V (range: 25 V – 60 V; Fig. 1) until aversive responses to electrical nociceptive stimulation were noticed. All eight cows in the NBA group, and four of the eight cows in the IVRA group expressed no aversive reaction to the cut-off level of 150 V fifteen min after placement of the local anaesthetic (four cows responded at 75 V, 80 V, 90 V, and 100 V, respectively) (LA) (Fisher’s EXACT Test: 8 NBA cows vs. 4 IVRA cows with no response: *p* = 0.077). No cows responded to electrical stimulation at the 150 V level 30 min after anaesthesia. The ANOVA revealed significant effects for group (*p* = 0.033) and time (*p* < 0.001; group x time: *p* = 0.019).

After removing the adhesive electrodes from the coronary band macroscopically, no skin alterations were detected except for an adelomorphic hyperaemia of the skin in all cows, which completely disappeared within 24 h after stimulation.

##### Thermal nociceptive stimulation

Nine of the 16 cows showed no aversive response at the safety cut-off level of 55 °C upon thermal stimulation before LA. Four of the remaining cows responded to thermal stimulation at 47 °C, 49 °C, 50 °C, 51 °C, and three cows responded at 52 °C. No cows responded at the safety level of 55 °C 15 min and 30 min after LA. Before anaesthesia, over 50% of the tested cows showed no response to maximal thermal stimulation; therefore, these results were not used for further statistical evaluation.

After removing the thermal probe from the coronary band, the skin area was reddened but without obvious swelling or exsudation. The next day, the skin reddening disappeared almost completely, and swelling and exudation were not obvious, but a very thin, dry, transparent, corrugated tissue layer separated from the skin was observed. The alteration was macroscopically diagnosed as a desquamated superficial corneal layer of the epidermis.

##### Mechanical nociceptive stimulation

Before LA application 7, 10, 13, 14, 13, and 14 cows of the 16 cows had no response to mechanical nociceptive stimulation at the skin of the lateral and medial heel bulb, the skin lateral and medial to the flexor tendons and dorsal at the lateral and medial first phalanx, respectively, at the cut-off level of 20 N. Cows responding to mechanical stimulation reacted at the different sites between 14 and 19 N. No cow responded at the safety cut-off level of 20 N at any site 15 min and 30 min after LA. No further statistical evaluation of these results was performed as a high proportion of cows revealed no aversive response to mechanical nociceptive stimulation prior to anaesthesia. No skin alterations were apparent macroscopically after mechanical stimulation.

#### Stress responses to changes in body position

Changing the cow’s body position from standing to LR on a surgical tipping table resulted in significantly increased blood concentrations of mean cortisol (*p* < 0.001), lactate (p < 0.001), NEFA (*p* = 0.038), and in the HR (*p* = 0.002), while blood glucose concentrations, RR, and MAP remained statistically unaffected (Table 1). No statistically significant group differences or group*time interactions were found.

**Table 1:** Hormonal-metabolic (blood concentrations of cortisol, lactate, glucose, and NEFA) and cardio-respiratory stress responses (HR, RR, MAP; mean ± SEM) to lateral recumbency (LR) restraint in dairy cows (before local anaesthesia in a distal hind distal limb; IVRA: intra venous regional anaesthesia, *N* = 8; NBA: hindfoot nerve block anaesthesia, N = 8; t-20: 20 min before, t-5: five min before, t5: five min after restraining the cows)

		Time	Relative	To	LR		(Minutes)	Statistics		(p-values)
		t-20		t-5		t5				
Parameter	Group	Mean	SEM	Mean	SEM	Mean	SEM	Group	Time	Group*time
Cortisol	IVRA	20.7	3.0	18.0	2.2	**43.2**	5.2	0.19	< 0.001	0.70
(ng/ml)	NBA	17.9		10.7		**37.2**				
Lactate	IVRA	2.04	0.20	2.04	0.23	**2.61**	0.30	0.31	< 0.001	0.98
(mmol/l)	NBA	2.34		2.36		**2.93**				
Glucose	IVRA	3.90	0.13	3.91	0.15	3.81	0.09	0.14	0.30	0.91
(mmol/l)	NBA	4.16		4.13		3.99				
NEFA	IVRA	262	45	275	49	**318**	70	0.74	0.038	0.89
(μmol/l)	NBA	231		250		**298**				
HR	IVRA	62	4	63	5	**70**	6	0.12	0.002	0.54
(1/min)	NBA	71		71		**83**				
RR	IVRA	28	3	30	3	31	3	0.59	0.51	0.46
(1/min)	NBA	27		30		26				
MAP	IVRA	142	5	145	5	141	7	0.76	0.75	0.37
(mmHg)	NBA	143		143		149				

Bold mean values are significantly different (p < 0.05) from the baseline values within groups

*NEFA* non-esterified fatty acids, *HR* heart rate, *RR* respiratory rate, *MAP* mean arterial blood pressure

#### Stress response to local anaesthesia

After administering LA, a significant increase occurred in the mean blood concentrations of cortisol (time effects: p < 0.001), lactate (*p* = 0.013), glucose (p < 0.001), and NEFA (*p* = 0.001), and in HR (*p* = 0.005), RR (p < 0.001), and MAP (*p* = 0.016; Table 2). No statistically significant group effects were found, but the RR revealed a group*time interaction of *p* = 0.016. Results revealed a significant increase in mean RR after IVRA while RR remained almost unchanged after NBA.

**Table 2:** Hormonal-metabolic (blood concentrations of cortisol, lactate, glucose, and NEFA) and cardio-respiratory stress responses (HR, RR, MAP; mean ± SEM) to local anaesthesia in the distal hind limb (IVRA: intravenous regional anaesthesia, N = 8; NBA: hindfoot nerve block anaesthesia, N = 8; t-5: 5 min before, t2, t14, t29, t40: 2, 14, 29, and 40 min after anaesthesia)

		Time	Relative	To	Anesthesia		(Minutes)					Statistics		(p-values)
		t-5		t2		t14		t29		t40				
Parameter	Group	Mean	SEM	Mean	SEM	Mean	SEM	Mean	SEM	Mean	SEM	Group	Time	Group*time
Cortisol	IVRA	48.7	6.6	53.5	6.9	**65.4**	7.0	**73.3**	7.0	**77.4**	6.3	0.90	< 0.001	0.17
(ng/ml)	NBA	43.1		**61.9**		**62.8**		**69.3**		**81.6**				
Lactate	IVRA	2.99	0.54	**4.36**	0.76	**5.02**	0.78	**5.57**	0.99	**5.60**	1.02	0.80	0.013	0.34
(mmol/l)	NBA	3.76		4.05		4.01		4.77		5.65				
Glucose	IVRA	3.81	0.14	4.01	0.11	4.06	0.16	4.48	0.25	**4.84**	0.30	0.66	< 0.001	0.78
(mmol/l)	NBA	3.98		**4.21**		4.18		**4.46**		**4.86**				
NEFA	IVRA	324	69	321	72	346	91	**397**	81	**451**	84	0.70	0.001	0.14
(μmol/l)	NBA	308		320		308		328		358				
HR	IVRA	73	6	**83**	7	68	6	**84**	8	**84**	7	0.53	0.019	0.44
(1/min)	NBA	76		**88**		82		**83**		**88**				
RR	IVRA	23	3	**32**	4	**29.8**	3.1	**33.0**	3.2	**36.3**	4	0.90	< 0.001	0.016
(1/min)	NBA	29		29		27.8		**35.3**		31.8				
MAP	IVRA	137	5	**148**	5	**148**	4	145	4	**146**	4	0.35	0.016	0.47
(mmHg)	NBA	146		148		151		149		154				

Bold mean values indicate significant differences (p < 0.05) in mean values within groups compared to baseline values

*NEFA* non-esterified fatty acids, *HR* heart rate, *RR* respiratory rate, *MAP* mean arterial blood pressure

#### Stress response to removal of tourniquet

The IVRA tourniquet was removed from the metatarsus after cows were returned from LR to standing. In NBA cows no tourniquet was used. Mean blood concentrations of cortisol, lactate, and NEFA as well as HR, RR, and MAP significantly decreased over time, while blood glucose concentrations continued to increase (Table 3). Statistical analysis revealed significant group*time interactions for NEFA (*p* = 0.015), and MAP (*p* = 0.010), and a group effect for MAP (*p* = 0.004). Before removal of the tourniquet, mean serum NEFA concentrations (*p* < 0.05), and before and after tourniquet removal MAP (p < 0.05) were greater in IVRA compared to NBA cows.

**Table 3:** Hormonal-metabolic (blood concentrations of cortisol, lactate, glucose, and NEFA) and cardio-respiratory stress responses (HR, RR, MAP; mean ± SEM) in standing cows to removal of the metatarsal tourniquet used for IVRA in a hind limb (intravenous regional anaesthesia, N = 8)

		Time	Relative	To	Tourniquet	Removal	(Min)	Statistics		(p-values)
		t-5		t10		t20				
Parameter	Group	Mean	SEM	Mean	SEM	Mean	SEM	Group	Time	Group*time
Cortisol	IVRA	76.2	6.8	72.7	7.0	**62.9**	7.2	0.83	0.002	0.84
(ng/ml)	NBA	72.9		71.7		61.0				
Lactate	IVRA	5.71	0.94	**5.02**	0.85	**3.52**	0.61	0.82	< 0.001	0.27
(mmol/l)	NBA	5.14		**4.70**		**3.63**				
Glucose	IVRA	5.17	0.34	5.17	0.32	5.35	0.35	0.49	0.017	0.32
(mmol/l)	NBA	5.34		**5.53**		5.81				
NEFA	IVRA	481^**a**^	62	435	59	**315**	44	0.11	< 0.001	0.015
(μmol/l)	NBA	297^**b**^		293		**246**				
HR	IVRA	84	8	86	6	80	6	0.97	0.088	0.16
(1/min)	NBA	95		**79**		**76**				
RR	IVRA	39.5	4.1	34.8	5.2	**30.0**	3.2	0.34	0.007	0.71
(1/min)	NBA	32.0		30.8		25.3				
MAP	IVRA	168^**a**^	4	**151** ^**a**^	3	**146** ^**a**^	3	0.004	< 0.001	0.010
(mmHg)	NBA	142^**b**^		142^**b**^		135^**b**^				

Recordings at corresponding time points in cows after NBA in which no tourniquet was used (hindfoot nerve block anaesthesia, N = 8; t-5: 5 min before, and t10, t20: 10 and 20 min after tourniquet removal in IVRA cows and at corresponding time points in NBA cows). Significant differences (p < 0.05) between corresponding group mean values at specific time points are indicated by different superscript letters (a,b). Bold mean values indicate significant differences (p < 0.05) in mean values within groups compared to baseline values

*NEFA* non-esterified fatty acids, *HR* heart rate, *RR* respiratory rate, *MAP* mean arterial blood pressure

### Study 2

#### Electrical nociceptive stimulation

**Fig. 2:**
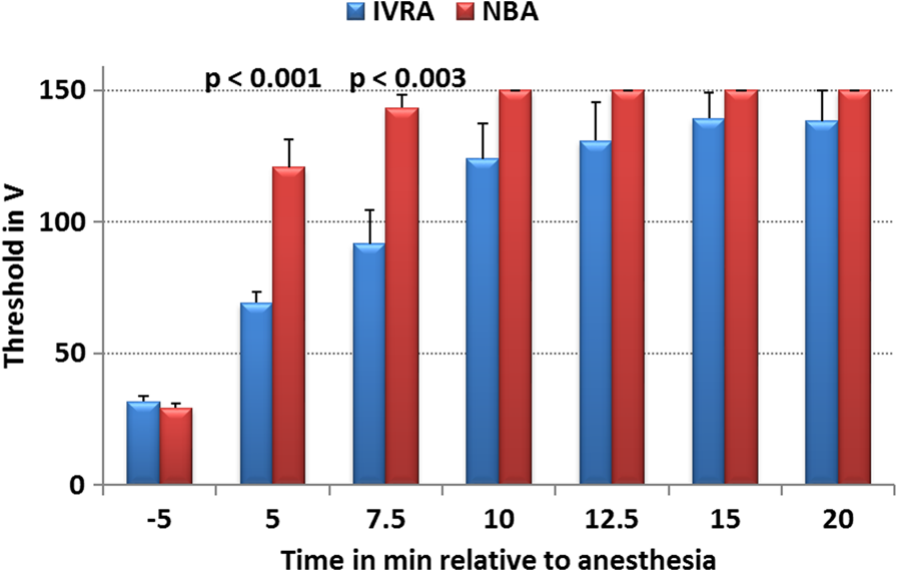
Mean thresholds (mean ± SEM) of electrical nociceptive stimulation at the lateral coronary band of the lateral claw of the right hind limb until an aversion response was noticed in dairy cows before (t-5 min) and after intravenous regional anaesthesia (IVRA; *N* = 6) and hindfoot nerve block anaesthesia (NBA; N = 6; study 2). Safety cut-off of electrical stimulation was 150 V (ANOVA effects: group: *p* = 0.018, time: p < 0.001, group x time: p < 0.001; significant group differences in mean values at specific time points are indicated by *p*-values within the graph)

**Fig. 3:**
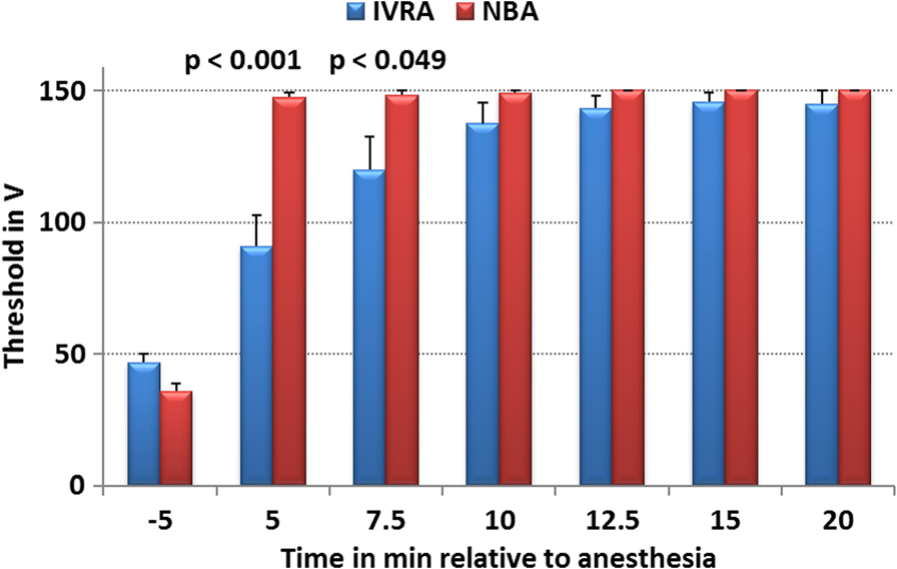
Thresholds (mean ± SEM) of electrical nociceptive stimulation at the heel bulb skin of the lateral claw of the right hind limb until an aversion response was noticed in dairy cows before (t-5 min) and after intravenous regional anaesthesia (IVRA; N = 6) and hindfoot nerve block anaesthesia (NBA; N = 6; study 2). Safety cut-off of electrical stimulation was 150 V (ANOVA effects: group: *p* = 0.014, time: p < 0.001, group x time: p < 0.001; significant group differences in mean values at specific time points are indicated by p-values within the graph)

Before LA, mean thresholds of electrical nociceptive stimulation at the dorso-lateral coronary band was 30.4 ± 4.3 V (range: 25–40 V). At the heel bulb skin, aversive responses to electrical nociceptive stimulation were registered after exceeding a mean threshold of 41.2 ± 8.7 V (range: 25–60 V; p = 0.015; Figs. 2 and 3). Mean thresholds of electrical stimulation at the dorso-lateral coronary band in cows from study 1 (Fig. 1) and study 2 (Fig. 2) were not significantly different (*p* = 0.32) before LA.

At the dorso-lateral coronary band (time effect: *p* < 0.001), as well as at the heel bulb skin, a steady increase in mean electrical stimulation thresholds was recorded after LA (time effect: p < 0.001). After NBA, the recorded increase in mean thresholds at the coronary band (ANOVA group effect: *p* = 0.018, group x time effect: p < 0.001) and at the heel skin (ANOVA group effect: *p* = 0.014, group x time effect: *p* = 0.001) were faster than those of IVRA (Figs. 2 and 3).

At the coronary band, no cows in the NBA group showed an aversive reaction 10 min after anaesthesia when stimulated at the cut-off level of 150 V, while 4 of the 6 cows in the IVRA group responded (responses at 65 V, 120 V, 120 V, and 140 V). At 20 min after anaesthesia, one IVRA cow responded to electrical stimulation at 80 V (Fisher’s EXACT test: group effect: *p* = 0.008).

At the heel bulb skin 10 min after NBA, no cows responded to the cut-off stimulation of 150 V, except one who responded at 140 V. The last cow in the NBA group reached the cut-off level at 12.5 min after LA. Ten minutes after IVRA, three of six cows responded to 100 V, 130 V, and 140 V stimulation. Twenty min after IVRA, one cow responded to 120 V stimulation (Fisher’s EXACT test: group effect: p < 0.001).

## Discussion

Intravenous regional anaesthesia [9, 18, 19, 36] and hindfoot nerve blocks [28] are long-standing recommended techniques for anaesthesia of the bovine digit. Onset of anaesthesia occurs approximately five minutes after IVRA and is optimal after ten minutes for major surgeries [20, 21, 26, 27, 37]. However, our study found that not all cows were completely desensitized to electrical stimulation of the distal limb at 15 min (study 1) and 20 min (study 2) after IVRA. In contrast, all cows in the NBA group were completely desensitized after approximately 12 min based on electrical stimulation results. This relatively slow onset of anaesthesia after IVRA contradicts previous reports and may be relevant in the field when major surgeries are performed under time constraints. In this study, only healthy animals were used. However, when anaesthesia of the distal limb is required for major surgeries of the claw, the affected distal limb frequently exhibits infectious cellulitis [9]. Onset and quality of IVRA and NBA in infectious, hyperaemic, and inflamed limbs may deviate from these results. In this study, 20 ml of procaine 2% was used for IVRA. Higher injection volumes [21, 27, 38] of procaine may expedite the desensitization process after IVRA. Other local anaesthetics, or concentrations higher than 2%, may also result in faster desensitization of the distal limb. This study used the common dorsal digital vein for procaine injections; however, use of the medial or lateral common plantar digital veins may reduce anaesthesia onset times. Before IVRA is used in humans, exsanguination is achieved using pneumatic tourniquets and lifting the treated extremity [24, 39, 40]. In this study, blood was drained from the punctured vein under gentle flexion of the fetlock joint, which is likely less effective in clearing space for local anaesthetics within the vascular system than the use of pneumatic tourniquets. However, options for improving IVRA efficacy have not been tested in cattle.

A valid instrument for demonstrating pain should produce a consistent response, show a correlation between stimulus intensity and pain and be easily interpreted by the observer. It should be easy to apply and have a clear cut-off point, while not damaging the tissue [41]. Electrical, mechanical and thermal nociceptive tests are validated in various species and commonly applied [41–46]. Electrical stimuli activate nonspecific peripheral fibres, including large-diameter fibres not directly involved in nociception [42]. Mechanical stimuli generate action potentials due to ionic influx in channels activated by stretch, and thermal stimuli involve membrane proteins or intracellular effector molecules with high temperature coefficients [43]. Because electrical stimulation is seen as a nonspecific nociceptive test, thermal and mechanical stimulation were also used to evaluate efficacy of distal limb desensitization after LA; however, a high proportion of cows did not respond to thermal or mechanical stimuli at the chosen cut-off levels before LA. Accordingly, mechanical and thermal stimulation appears unsuitable to detect nociception in cows restrained on a tipping table in LR.

Luna et al. [41] validated electrical, mechanical and thermal stimulation and found all techniques were valid for assessing nociception in horses. Mechanical and thermal stimulation were performed using wireless devices while the horses were free and undisturbed in their stalls. The authors reported a thermal stimulation response at an average of 51 °C at the limb and 55 °C at the thorax using a safety cut-off level of 60 °C. Most horses responded to thermal stimulation prior to the cut-off. The reported thermal thresholds were close to or at the safety cut-off level of 55 °C used in this study. Ambient temperature influences the outcome of thermal nociceptive testing [44]. In horses, thermal stimulation at higher ambient temperatures (> 20 °C: 53 ± 4 °C) resulted in greater thresholds until a response occurred compared to low ambient temperatures (10 °C; 49 ± 6 °C) [31]. Performing thermal nociceptive stimulation at room temperature (21 °C) on the cows in this study may have contributed to the high frequency of non-responders at the cut-off level. It appears that a cut-off level of 60 °C would have reduced the number of non-responders to thermal stimulation in this study. However, it is important to consider that all cows exhibited superficial skin alterations at the coronary band after thermal stimulation using the 55 °C cut-off level. Before higher cut-off levels for thermal stimulation are used, more studies are needed to evaluate the degree of tissue damage from heat stimulation at different cut-off levels and anatomic locations after single and repeated stimulations.

In this study, using a probe tip of 1.7 mm diameter and a safety cut-off of 20 N, most cows did not respond to mechanical stimulation at the distal limb even before LA. In contrast, unrestrained horses always responded to mechanical stimulation at the metacarpus using a wireless device with a 1 mm probe. The average nociceptive threshold at which the horses responded was 3–4 N [41]. Recently, Raundal et al. [45] compared two handheld devices for mechanical stimulation, a 0.8 mm vs. 6.5 mm diameter probe, at the metatarsus of unrestrained cattle. On average cows reacted aversively to both the 0.8 mm and 6.5 mm probes at 4 N and at 45 N, respectively. Other authors have reported mean mechanical nociceptive stimulation thresholds at the coronary band in cattle to be 14 N using a 2.5 mm probe [46], and 9 N using a 2 mm probe, at the dorsal aspect of the metatarsus [47]. Thus, it is unlikely that the stoic nature of cows [13] explains the high frequency of non-responders to mechanical nociceptive stimulation in this study.

Restraining cows in LR on a hydraulic surgical tipping table allowed safe LA application. Artificial isolation from the herd, close human-cow contact, a novel environment and restraint induce stress response [15, 34] as indicated by increased blood concentrations of cortisol, lactate, and NEFA, and by an increased HR. LR also impairs respiration in cows leading to a moderate increase in arterial pCO_2_ and a decrease in pO_2_. This impairment, together with the adrenergic stress response, is reflected here and in previous studies [15, 34] by an increase in blood lactate levels caused by anaerobic glycolysis in peripheral tissues. Clinical pain is a complex mechanism involving sensory-discriminative, cognitive and emotional aspects [41]. An increase in nociceptive thresholds after exposure to acute stressors (stress induced hypo- or analgesia: SIA) [44] has been found in various species [48, 49] including cattle [50, 51]. It is likely that LR-induced SIA contributed to the cows’ unresponsiveness to mechanical and thermal nociceptive stimulation. Accordingly, needle pricks to the interdigital space as a common nociceptive test for foot desensitization [38] after LA and before surgical interventions should be reconsidered. Major claw surgeries are common on restrained and sick cows. Thus, SIA may also modulate needle prick results, providing inaccurate information on the degree of desensitization after LA.

Administering IVRA requires placing a tourniquet. Human patients commonly experience tourniquet pain within 30 min after placement [39, 52–54]. In this study, mean hormonal-metabolic and cardiac stress responses to IVRA and NBA in cows, were of similar magnitude (except the respiratory rate), giving no clear indication of specific pain-related stress responses by either technique. However, pain-induced stress responses may have been masked by stress from the LR. In the IVRA group the tourniquet was removed from the metatarsus after returning the cows back to standing position. The significantly higher mean blood NEFA concentration and MAP in the IVRA cows before and after removing the tourniquet compared to those of the NBA group may indicate a stress response induced by tourniquet pain.

## Conclusion

Despite the small sample number in this study, the results from the electrical nociceptive stimulation indicate that intravenous regional anaesthesia and hindfoot four-point nerve blocks completely desensitize the distal limb in dairy cows. However, complete desensitization developed significantly faster after NBA (12 min) than after IVRA (≥ 20 min). This study found no specific stress responses to placement of the tourniquet, but they may have been masked by the responses of the animals to restraint in lateral recumbency. The time difference until complete desensitization between both LA techniques in this study appears clinically relevant, as distal limb anaesthesia is often necessary for major claw surgeries under time constraints in the field.

## Abbreviations

HF: Holstein Friesian
HR: Heart rate
IVRA: Intravenous regional anaesthesia
LA: Local anaesthesia
LR: Lateral recumbency
MAP: Mean arterial blood pressure
NBA: hindfoot four-point nerve block anaesthesia
NEFA: Non-esterified fatty acids
RR: Respiratory rate

## References

[1] Murray RD, Downham DY, Clarkson MJ, Faull WB, Hughes JW, Manson FJ, Merritt JB, Russell WB, Sutherst JE, Ward WR (1996). Epidemiology of lameness in dairy cattle: description and analysis of foot lesions. Vet Rec.

[2] Barker ZE, Leach KA, Whay HR, Bell NJ, Main DC (2010). Assessment of lameness prevalence and associated risk factors in dairy herds in England and Wales. J Dairy Sci.

[3] Main DC, Barker ZE, Leach KA, Bell NJ, Whay HR, Browne WJ (2010). Sampling strategies for monitoring lameness in dairy cattle. J Dairy Sci.

[4] Solano L, Barkema HW, Mason S, Pajor EA, LeBlanc SJ, Orsel K (2016). Prevalence and distribution of foot lesions in dairy cattle in Alberta Canada. J Dairy Sci.

[5] Cook NB, Hess JP, Foy MR, Bennett TB, Brotzman RL (2016). Management characteristics, lameness, and body injuries of dairy cattle housed in high-performance dairy herds in Wisconsin. J Dairy Sci.

[6] Thomas HJ, Miguel-Pacheco GG, Bollard NJ, Archer SC, Bell NJ, Mason C, Maxwell OJ, Remnant JG, Sleeman P, Whay HR (2015). Evaluation of treatments for claw horn lesions in dairy cows in a randomized controlled trial. J Dairy Sci.

[7] Thomas HJ, Remnant JG, Bollard NJ, Burrows A, Whay HR, Bell NJ, Mason C, Huxley JN (2016). Recovery of chronically lame dairy cows following treatment for claw horn lesions: a randomised controlled trial. Vet Rec.

[8] Toussaint Raven E (2003). Cattle Footcare and claw trimming.

[9] Heppelmann M, Kofler J, Meyer H, Rehage J, Starke A (2009). Advances in surgical treatment of septic arthritis of the distal interphalangeal joint in cattle: a review. Vet J.

[10] Starke A, Heppelmann M, Beyerbach M, Rehage J (2007). Septic arthritis of the distal interphalangeal joint in cattle: comparison of digital amputation and joint resection by solar approach. Vet Surg.

[11] Offinger J, Herdtweck S, Rizk A, Starke A, Heppelmann M, Meyer H, Janssen S, Beyerbach M, Rehage J (2013). Postoperative analgesic efficacy of meloxicam in lame dairy cows undergoing resection of the distal interphalangeal joint. J Dairy Sci.

[12] Desrochers A, St Jean G (1996). Surgical management of digit disorders in cattle. Vet Clin Food Anim.

[13] Anderson DE, Edmondson MA (2013). Prevention and management of surgical pain in cattle. Vet Clin Food Anim.

[14] Edmondson MA (2008). Local and regional anesthesia in cattle. Vet Clin Food Anim.

[15] Rizk A, Herdtweck S, Offinger J, Meyer H, Zaghloul A, Rehage J (2012). The use of xylazine hydrochloride in an analgesic protocol for claw treatment of lame dairy cows in lateral recumbency on a surgical tipping table. Vet J.

[16] Janssen S, Wunderlich C, Heppelmann M, Palme R, Starke A, Kehler W, Steiner A, Rizk A, Meyer U, Daenicke S (2016). Short communication: pilot study on hormonal, metabolic, and behavioral stress response to treatment of claw horn lesions in acutely lame dairy cows. J Dairy Sci.

[17] Bier A (1908). A new way to induce local anesthesia in the distal limb (Über einen neuen Weg Lokalanästhesie an den Gliedmaßen zu erzeugen). Arc Klin Chir.

[18] Antalovsky A (1965). Technika mistni nitrozilni anestezie na distalnich castech koncetin u skotu (technique of intravenous local anesthesia in the distal limb in cattle). Vet Med.

[19] Avemann M (1974). Prüfung des von ANTALOVSKY angegebenen Verfahrens zur intravenösen regionalen Betäubung im Zehenbereich des Rindes auf seine Brauchbarkeit (usefulness of intravenenous regional anaesthesia according to ANTALOVSKY in the distal limb of cattle).

[20] Maierl J, Nuss K, Fiedler A, Maierl J (2004). Anatomische Grundlagen und Lokalanästhesie. Nuss K: Erkankungen der Klauen und der Zehen des Rindes.

[21] Greenough PR: Regional anesthesia, regional antibiotic perfusion, and nerve blocks. In: Geenough PR: Bovine laminitis and lameness. A hands-on approach. London: Saunders Elsevier; 2007. p.252.

[22] Potterton SL, Bell NJ, Whay HR, Berry EA, Atkinson OC, Dean RS, Main DC, Huxley JN (2012). A descriptive review of the peer and non-peer reviewed literature on the treatment and prevention of foot lameness in cattle published between 2000 and 2011. Vet J.

[23] Halladin NL, Zahle FV, Rosenberg J, Gogenur I (2014). Interventions to reduce tourniquet-related ischaemic damage in orthopaedic surgery: a qualitative systematic review of randomised trials. Anaesthesia.

[24] Estebe JP, Davies JM, Richebe P (2011). The pneumatic tourniquet: mechanical, ischaemia-reperfusion and systemic effects. Europ J Anaes.

[25] Copland VS, Hildebrand SV, Hill T, Wong P, Brock N (1989). Blood pressure response to tourniquet use in anesthetized horses. JAVMA.

[26] Skarda R: Local and Regional Anesthetic Techniques: Ruminants and Swine. In Thurmon JC, Tranquilli WJ, Benson GJ, editors. Lumb & Jones' Veterinary Anesthesia. *Baltimore: Williams & Wilkins, A Waverly Company; 1996. p. 479–514*.

[27] Skarda RT: Local and regional analgesia. In: Short CE, editor. Principles & practice of veterinary anesthesia**.** William & Wilkens, Baltimore*,* USA*;* 1987. *P.* 91*–*133, ISBN 0-683-07702-3.

[28] Raker CW (1956). Regional anesthesia of the bovine foot. JAVMA.

[29] Levionnois OL, Spadavecchia C, Kronen PW, Schatzmann U (2009). Determination of the minimum alveolar concentration of isoflurane in Shetland ponies using constant current or constant voltage electrical stimulation. Vet Anaesth Analg.

[30] Hopster K, Muller C, Hopster-Iversen C, Stahl J, Rohn K, Kastner S (2014). Effects of dexmedetomidine and xylazine on cardiovascular function during total intravenous anaesthesia with midazolam and ketamine and recovery quality and duration in horses. Vet Anaesth Analg.

[31] Poller C, Hopster K, Rohn K, Kastner SB (2013). Evaluation of contact heat thermal threshold testing for standardized assessment of cutaneous nociception in horses - comparison of different locations and environmental conditions. BMC Vet Res.

[32] Poller C, Hopster K, Rohn K, Kastner SB (2013). Nociceptive thermal threshold testing in horses - effect of neuroleptic sedation and neuroleptanalgesia at different stimulation sites. BMC Vet Res.

[33] Schütter AF, Tunsmeyer J, Kastner SB: Influence of metamizole on 1) minimal alveolar concentration of sevoflurane in dogs and 2) on thermal and mechanical nociception in conscious dogs. Vet Anaesth Analg 2016*,* 43*:* 215*–*226*.* 10.1111/vaa.12289 26234314

[34] Rizk A, Herdtweck S, Meyer H, Offinger J, Zaghloul A, Rehage J (2012). Effects of xylazine hydrochloride on hormonal, metabolic, and cardiorespiratory stress responses to lateral recumbency and claw trimming in dairy cows. JAVMA.

[35] Offinger J, Meyer H, Fischer J, Kastner SB, Piechotta M, Rehage J (2012). Comparison of isoflurane inhalation anaesthesia, injection anaesthesia and high volume caudal epidural anaesthesia for umbilical surgery in calves; metabolic, endocrine and cardiopulmonary effects. Vet Anaesth Analg.

[36] Prentice DE, Wyn-Jones GW, Jones RS, Jagger DW (1974). Intravenous regional anaesthesia of the bovine foot. Vet Rec.

[37] Trim CM: Special Anesthesia Considerations in the Ruminant. *in Short CE, editor: Principles and Practice of Veterinary Anesthesia. Baltimore: Williams & Williams;* 1987. p.285–308. ISBN 0–683–07702-3:285–308.

[38] Hudson C, Whay H, Huxley J: Recognition and management of pain in cattle. *In Practice (0263841X)* 2008, 30(3):126–134.

[39] Rodola F, Vagnoni S, Ingletti S (2003). An update on intravenous regional Anaesthesia of the arm. Eur Rev Med Pharmaco.

[40] Flamer D, Peng PW (2011). Intravenous regional anesthesia: a review of common local anesthetic options and the use of opioids and muscle relaxants as adjuncts. Local Reg Anesth.

[41] Luna SP, Lopes C, Rosa AC, Oliveira FA, Crosignani N, Taylor PM, Pantoja JC (2015). Validation of mechanical, electrical and thermal nociceptive stimulation methods in horses. Equine Vet J.

[42] Le Bars D, Gozariu M, Cadden SW (2001). Animal models of nociception. Pharmacol Rev.

[43] Neddermeyer TJ, Fluhr K, Lotsch J (2008). Principle components analysis of pain thresholds to thermal, electrical, and mechanical stimuli suggests a predominant common source of variance. Pain.

[44] Love EJ, Murrell J, Whay HR (2011). Thermal and mechanical nociceptive threshold testing in horses: a review. Vet Anaesth Analg.

[45] Raundal PM, Andersen PH, Toft N, Forkman B, Munksgaard L, Herskin MS (2014). Handheld mechanical nociceptive threshold testing in dairy cows - intra-individual variation, inter-observer agreement and variation over time. Vet Anaesth Analg.

[46] Tadich N, Tejeda C, Bastias S, Rosenfeld C, Green LE (2013). Nociceptive threshold, blood constituents and physiological values in 213 cows with locomotion scores ranging from normal to severely lame. Vet J.

[47] Whay HR, Webster AJ, Waterman-Pearson AE (2005). Role of ketoprofen in the modulation of hyperalgesia associated with lameness in dairy cattle. Vet Rec.

[48] Rushen J, Ladewig J (1991). Stress-induced hypoalgesia and opioid inhibition of pigs' responses to restraint. Physiol Behav.

[49] Cook CJ, Maasland SA, Devine CE (1996). Social behaviour in sheep relates to behaviour and neurotransmitter responses to nociceptive stimuli. Physiol Behav.

[50] Rushen J, Boissy A, Terlouw EM, de Passille AM (1999). Opioid peptides and behavioral and physiological responses of dairy cows to social isolation in unfamiliar surroundings. J Anim Sci.

[51] Herskin MS, Munksgaard L, Ladewig J (2004). Effects of acute stressors on nociception, adrenocortical responses and behavior of dairy cows. Physiol Behav.

[52] Chiao FB, Chen J, Lesser JB, Resta-Flarer F, Bennett H (2013). Single-cuff forearm tourniquet in intravenous regional anaesthesia results in less pain and fewer sedation requirements than upper arm tourniquet. Brit J Anaesth.

[53] Kam PC, Kavanagh R, Yoong FF (2001). The arterial tourniquet: pathophysiological consequences and anaesthetic implications. Anaesthesia.

[54] Choyce A, Peng P (2002). A systematic review of adjuncts for intravenous regional anesthesia for surgical procedures. Can J Anaesth.

